# Surgical Outcomes and Margin Assessment by Resection Tool in Tongue Cancer: A Systematic Review

**DOI:** 10.7759/cureus.100636

**Published:** 2026-01-02

**Authors:** Rami Saade, Gibran Atwi, Mia Harb, Elias Keyrouz

**Affiliations:** 1 Department of Otolaryngology Head &amp; Neck Surgery, Lebanese American University Medical Center, Beirut, LBN

**Keywords:** co₂ laser, electrocautery, harmonic scalpel, surgical margins, tongue cancer, transoral surgery

## Abstract

This systematic review aimed to evaluate surgical margin status and related outcomes according to the tool used for tongue resection. A systematic search of PubMed, Embase, Scopus, and the Cochrane Library was conducted up to June 2025. Studies were included if they reported surgical outcomes in tongue cancer resections performed with a specified tool. Key outcomes included margin status, recurrence, thermal damage, functional recovery, and complications. Given insufficient subgroup-level data for meta-analysis, a qualitative synthesis was performed. Fifteen studies were included, comprising 582 patients across six primary resection tools. CO_2_ laser and transoral laser microsurgery (TLM) achieved consistently high rates of histologically negative margins but were associated with thermal artefacts that could impair epithelial interpretation, especially in irradiated fields. Harmonic scalpel and ultrasonic dissectors produced clear margins with minimal lateral tissue damage, though long-term oncologic outcomes were underreported. Cold steel provided artefact-free margins but was limited by greater intraoperative bleeding. Electrocautery yielded narrower margins and occasional interpretive artefacts but demonstrated short-term safety in selected patients. Functional outcomes, most robustly reported in TLM cohorts, indicated preserved speech and swallowing with low rates of long-term feeding tube or tracheostomy dependence. Complication rates were low across all modalities. Both thermal and non-thermal resection tools can achieve acceptable oncologic outcomes in tongue cancer surgery. However, margin clarity and interpretability vary substantially by modality. CO_2_ laser and ultrasonic/harmonic devices offer favorable profiles, but tool-specific differences in artefact and recurrence warrant further investigation in prospective comparative studies.

## Introduction and background

Achieving clear histopathological margins is a cornerstone of oncologic surgery in oral tongue squamous cell carcinoma (OTSCC), directly impacting local control, recurrence rates, and survival [1]. Despite this, margin positivity remains a frequent and clinically significant challenge, with reported rates ranging from 10% to 30% in contemporary series-even among experienced head and neck surgeons [2]. These positive margins are consistently associated with poorer disease-specific outcomes and increased need for adjuvant therapy, which may compound treatment-related morbidity [3]. Histopathological margins refer to the edges of excised tissue examined under a microscope to confirm whether cancer cells are present at the resection boundary. Achieving clear or “negative” margins indicates that the tumor has been completely removed. In contrast, “positive” margins signify residual disease and are strongly associated with higher recurrence risk. “Thermal artefacts” refer to microscopic tissue changes caused by heat from surgical instruments, which can distort cell morphology and complicate interpretation during pathology review [3].

While the importance of negative margins is well-established, the influence of the surgical tool used to achieve those margins has received comparatively limited scrutiny. A wide array of resection tools is available in clinical practice, including cold steel scalpel [4], electrocautery [5], CO_2_ laser [6], harmonic scalpel [7], and ultrasonic dissectors, each with distinct thermal properties, dissection mechanics, and hemostatic profiles [8,9]. These differences not only affect margin width but may also introduce artefacts that impair histopathological interpretability, especially when evaluating epithelial surfaces for dysplasia or carcinoma in situ. For example, laser- and cautery-based instruments often produce lateral thermal damage and pseudodysplastic epithelial changes [10], which can confound margin assessment and lead to diagnostic uncertainty. Despite the recognized importance of surgical margins, no prior systematic review has directly compared margin status and histological interpretability according to the resection tool used in tongue cancer. 

Moreover, newer modalities such as harmonic scalpel and ultrasonic dissection have been increasingly adopted in oncologic surgery due to their ability to balance precision with hemostasis [11]. However, their comparative performance in achieving oncologically sound and histologically interpretable margins in glossectomy has not been systematically evaluated. Most existing literature focuses on oncologic outcomes or surgical feasibility in isolation, without direct comparison across tool types or integration of functional and histopathologic considerations.

Given this variability, there is a pressing need to understand how the choice of surgical instrument affects margin status, recurrence risk, and downstream functional recovery. To our knowledge, no prior systematic review has synthesized the available evidence regarding margin adequacy and related outcomes in relation to the specific tool used for tongue resection. Addressing this gap is of both theoretical and practical significance, with implications for intraoperative decision-making, margin interpretation, and long-term treatment planning.

Accordingly, we conducted a systematic review to evaluate surgical margin status and associated outcomes (including local recurrence, thermal artefact, functional recovery, and postoperative complications) based on the tool used for tongue cancer resection. By synthesizing evidence across multiple resection technologies, this study aims to inform evidence-based tool selection and identify areas in need of further comparative research.

## Review

Methods

Study Design and Reporting Standards

This systematic review was conducted in accordance with the Preferred Reporting Items for Systematic Reviews and Meta-Analyses (PRISMA) 2020 guidelines [12]. The review protocol was prospectively developed but not registered, as no quantitative synthesis was planned a priori. The objective was to map and interpret the existing evidence based on surgical tools used for tongue cancer resection, which is a topic rarely analyzed separately, given that most oral cancer studies do not report tool-specific or site-stratified outcomes. All stages of study screening, selection, data extraction, and synthesis were independently performed by two reviewers, with discrepancies resolved by consensus. 

Search Strategy

A comprehensive search of MEDLINE (via PubMed), Scopus, and Web of Science was conducted from inception to July 8, 2025. The search strategy combined controlled vocabulary and free-text terms related to tongue cancer (e.g., “tongue neoplasms,” “oral tongue carcinoma”), resection tools (e.g., “CO_2_ laser,” “harmonic scalpel,” “cold steel,” “electrocautery”), and margin assessment (e.g., “surgical margins,” “deep margin,” “histologic clearance”). No language or date restrictions were applied. The complete search strategy for each database is available in the Appendices.

We also searched the Grey Literature (Google Scholar) as per recent guidelines [13]. Additionally, we performed a manual search of references through PubMed’s “similar article” function [14].

Eligibility Criteria

The eligibility criteria for inclusion were defined according to the population, intervention, comparison, outcomes and study (PICOS) framework [15]. Studies were considered eligible if they involved adult patients who underwent surgical resection for primary tongue cancer, regardless of the tumor stage. The intervention of interest was tumor resection performed using a clearly defined surgical tool, such as a CO_2_ laser, cold steel, harmonic scalpel, ultrasonic dissection device, or electrocautery. Eligible studies were required to report the surgical margin status and/or at least one relevant clinical outcome, including recurrence, functional recovery, or complication rates. The review included randomized controlled trials, cohort studies, case series with a minimum of five patients, and prospective observational studies. Only studies published in full text in English or German were considered.

Studies were excluded if they did not specify the surgical tool used for tumor resection. Additional exclusion criteria comprised animal studies, narrative reviews, conference abstracts, and expert opinion articles. Duplicate datasets or follow-up reports of previously published cohorts were also excluded unless they provided new or additional outcome data.

Study Selection

Titles and abstracts were screened independently by two reviewers. Full texts of potentially eligible articles were retrieved and reviewed in detail. Disagreements at any stage were resolved by consensus or third-party adjudication.

Data Extraction

From each included study, the following data were extracted: authorship, publication year, country of origin, study design, sample size, tumor site and stage, surgical tool used, method of margin assessment, margin status (positive, close, negative), recurrence rates, functional outcomes (e.g., feeding tube dependence, speech/swallowing recovery), and postoperative complications. Data were independently extracted by two reviewers using a standardized extraction form.

Risk of Bias Assessment

Methodological quality was assessed using the Newcastle-Ottawa Scale (NOS), which evaluates observational studies across three domains: selection of participants, comparability of study groups, and ascertainment of outcomes..

Data Synthesis and Justification for Non-Meta-Analysis

Given the diversity in outcome definitions, surgical tools, patient populations, and reporting metrics, quantitative synthesis was initially considered. However, subgroup-level pooling would require at least three studies per surgical tool type for each outcome of interest, which was not achieved for most outcome-tool combinations. Therefore, a meta-analysis was not feasible, and findings were summarized qualitatively and narratively by outcome domain (i.e., margin status, recurrence, functional outcomes, complications), stratified by the resection tool employed.

Results

Literature Search Results

**Figure 1:**
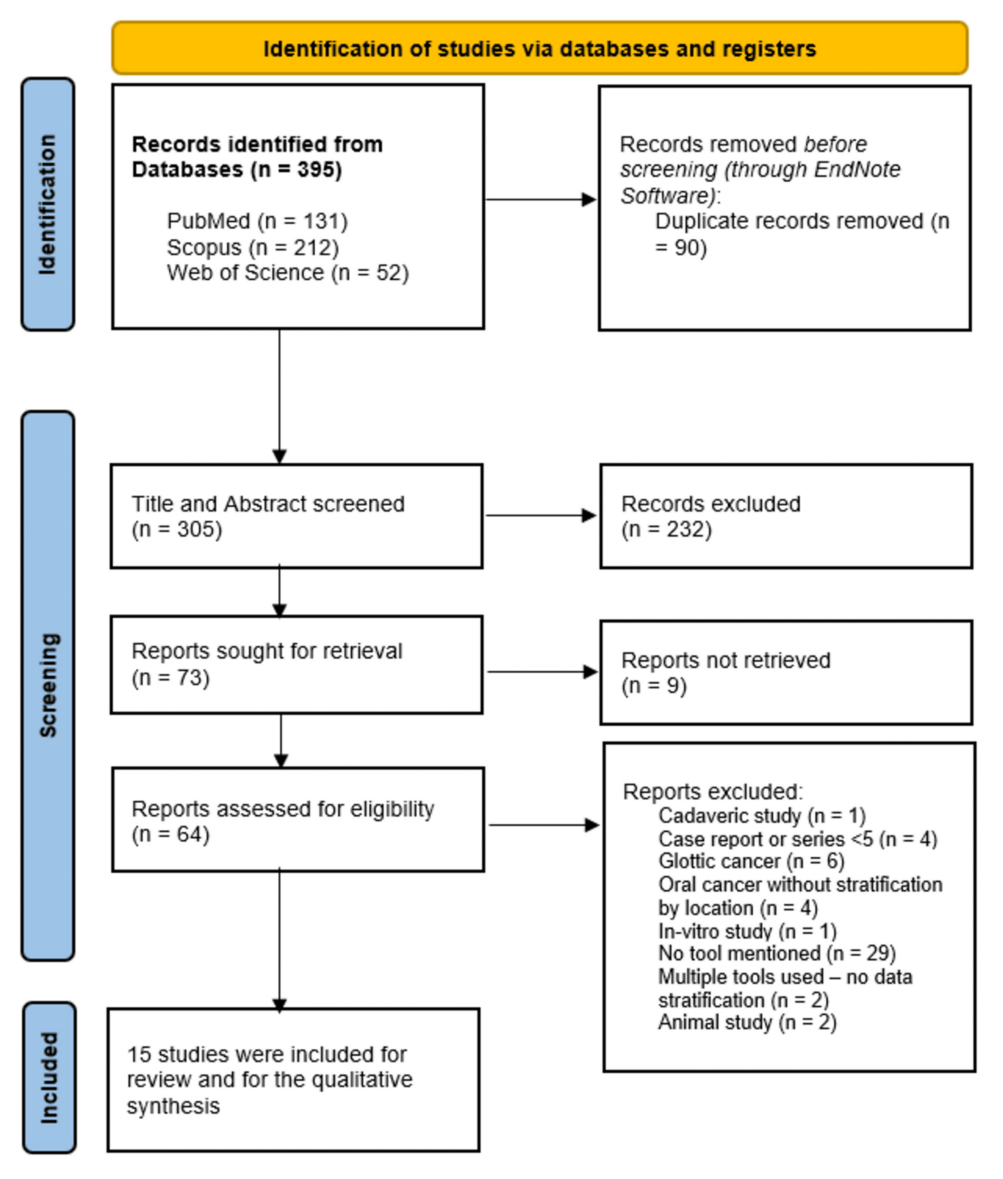
A PRISMA flow diagram showing the results of the database search

A total of 395 records were identified through systematic database searches, including PubMed (n = 131), Scopus (n = 212), and Web of Science (n = 52) (Figure 1). After removing 90 duplicate entries, 305 records underwent title and abstract screening. Of these, 232 records were excluded. The full texts of the remaining 73 articles were assessed for eligibility. A further 58 articles were excluded for the following reasons: cadaveric study (n = 1, 1.7%), case reports or small series with <5 patients (n = 4, 6.9%), studies on glottic cancer (n = 6, 10.3%), oral cancer studies without anatomical stratification to the tongue (n = 4, 6.9%), in-vitro study (n = 1, 1.7%), failure to mention the surgical tool used (n = 29, 50%), use of multiple tools without subgroup stratification (n = 2, 3.4%), animal study (n = 2, 3.4%), and full text not retrievable (n = 9, 15.5%). Ultimately, 15 studies met the inclusion criteria and were included in the qualitative synthesis [9,16-29].

Baseline Characteristics of Included Studies

Among included studies (Table 1), most evidence came from the United Kingdom (3 studies), followed by Italy (2 studies), while the remaining countries (i.e., Canada, China, Finland, Germany, and France) were represented by single studies. Most studies were retrospective cohort (13 studies), while one was a pilot comparative study, and the remaining one was a prospective series study. Overall, 615 patients were investigated, with Harmonic Focus Shears being the most frequently reported resection method (5 studies), followed by CO_2_ laser (4 studies), and monopolar electrocautery (2 studies). Other methods included monopolar resection, diathermy, transoral microsurgery, and YAG laser.

**Table 1:** Baseline characteristics of included studies YOP: year of publication; UK: United Kingdom; USA: United States of America

Author (YOP)	Country	Design	Group	Sample	Mean age	Male gender	Partial glossectomy	Excision margin (cm)
Carruth JA (1985) [16]	UK	Retrospective series	CO_2_ laser	100	-	-	10 (10%)	-
Cristalli et al. (2012) [18]	Italy	Pilot study	Monopolar electrocautery	11	-	-	10 (89.5%)	1.5
			Harmonic Focus Shears	8	-	-	-	1.5
Kimoto et al. (2017) [22]	Japan	Retrospective series	CO_2_ laser	31	65.8	18 (58.06%)	31 (100%)	-
Limongelli et al. (2020) [23]	Italy	Retrospective series	Diode laser (GaA1A)	85	62.81	50 (58.82%)	-	1
Rao et al. (2018) [27]	India	Retrospective series	Monopolar electrocautery	12	-	9 (75%)	-	-
			Ultrasonic coagulation device (Olympus)	12	-	9 (75%)	-	-
Choi et al. (2017) [17]	Korea	Retrospective series	Transoral bissected resection	30	49.3	17 (56.67%)	-	1.5
			Tansoral en bloc resection	45	53.9	25 (55.56%)	-	1.5
Luukkaa et al. (2002) [24]	Finland	Retrospective series	YAG laser	35	-	-	28 (80%)	-
Pons et al. (2009) [9]	France	Prospective series	Harmonic Focus Shears	18	61	15 (83.33%)	18 (100%)	-
			Monopolar resection	12	-	-	-	-
Tahim & Sadiq (2020) [28]	UK	Retrospective series	Harmonic Focus Shears	5	71.6	3 (60%)	5 (100%)	-
Nilsson et al. (2022) [26]	Sweden	Retrospective series	Monopolar diathermy	34	63	22 (65%)	-	1
			Harmonic Focus Shears	23	-	-	-	-
Frame et al. (1988) [19]	UK	Retrospective series	CO_2_ laser	21	62	10 (47.62%)	16 (76.19%)	-
Grant et al. (2006) [21]	USA	Retrospective series	Transoral microsurgery	59	65	35 (59%)	-	-
Metternich et al. (2002) [25]	Germany	Retrospective series	Harmonic	25	-	-	-	-
Wang et al. (2001) [29]	China	Retrospective series	CO_2_ laser	37	-	-	-	-
Gauthier et al. (2010) [20]	Canada	Retrospective series	Conventional scalpel	12	-	-	-	1.5

Methodological Quality

Using the Newcastle Ottawa Scale, four articles were deemed of good quality, six were of fair quality, and five were of poor quality (mainly due to small sample size and/or lack of information on follow-up period or losses to follow-up) (Table 2).

**Table 2:** Methodological quality of included studies using the Newcastle Ottawa Scale In the methodological quality assessment of included studies using the Newcastle–Ottawa Scale (NOS), a asterisk (*) indicates that the study meets the specific criterion, while a dash (-) indicates that it does not YOP: year of publication

Author (YOP)	Selection				Comparability	Outcome			Overall quality
	Exposure representativeness	Non-exposure selection	Exposure ascertainment	Outcome not prior to exposure	Design or analysis	Outcome assessment	Sufficient follow-up	Follow-up adequacy	
Carruth JA (1985) [16]	*	-	*	*	-	*	-	-	Fair
Cristalli et al. (2012) [18]	-	-	*	*	-	*	-	-	Poor
Kimoto et al. (2017) [22]	*	-	*	*	-	*	*	*	Good
Limongelli et al. (2020) [23]	*	-	*	*	-	*	*	*	Good
Rao et al. (2018) [27]	-	-	*	*	*	*	-	-	Poor
Choi et al. (2017) [17]	*	-	*	*	*	*	*	*	Good
Luukkaa et al. (2002) [24]	*	-	*	*	-	*	-	-	Fair
Pons et al. (2009) [9]	-	-	*	*	-	*	-	-	Poor
Tahim & Sadiq (2020) [28]	-	-	*	*	-	*	-	-	Poor
Nilsson et al. (2022) [26]	*	-	*	*	*	*	-	-	Fair
Frame et al. (1988) [19]	*	-	*	*	-	*	*	*	Good
Grant et al. (2006) [21]	*	-	*	*	-	*	-	-	Fair
Metternich et al. (2002) [25]	*	-	*	*	-	*	-	-	Fair
Wang et al. (2001) [29]	*	-	*	*	-	*	-	-	Fair
Gauthier et al. (2010) [20]	-	-	*	*	-	*	-	-	Poor

Margin Status

Margin status varied across studies and was closely associated with the surgical tool and technique employed (Table 3). Among studies utilizing CO_2_ laser for resection, histologically negative margins were consistently achieved, although interpretability was occasionally limited by thermal artefact. Carruth JA reported that all specimens excised with CO_2_ laser allowed for reliable histologic margin assessment, with no cases of postoperative hemorrhage or airway compromise [16]. Similarly, Kimoto et al. demonstrated a 90% rate (28 patients) of negative margins in 31 patients undergoing laser glossectomy for early-stage lesions, with only one instance requiring re-resection due to a positive margin [22]. Cristalli et al., however, reported a 17.9% rate (5 patients) of positive deep margins following CO_2_ laser resection, despite uniformly negative mucosal margins, highlighting the challenge of achieving depth control using transoral laser techniques [18].

**Table 3:** Summary of included studies assessing surgical margin status and oncologic outcomes according to tool of tongue resection R0: negative resection margin; TLM: transoral laser microsurgery; CO_2_: carbon dioxide; FS: frozen section; US: ultrasound; VAS: visual analog scale; FOSS: Functional Outcome Swallowing Scale; OR: operating room; Tx: treatment; RT: radiotherapy; CT: chemotherapy; DSS: disease-specific survival; LVI: lymphovascular invasion; TBR: transoral bisected resection; TER: transoral en bloc resection; PEG: percutaneous endoscopic gastrostomy; mo: month(s); wk: week(s); y: year(s); NA: not available

Study	Country	Tool of resection	Sample size	Margin status	Local recurrence	Path risk features	Oncologic outcomes	Functional outcomes	Death
Carruth JA (1985) [16]	UK	CO_2_ laser	87 (100 resections)	All margins assessable; no positive margins	None	Not reported	No recurrences reported	Minimal pain; no feeding tubes; early discharge	Not reported
Choi et al. (2017) [17]	South Korea	Bovie tip + Harmonic scalpel	75	TBR had wider deep margins (9.9 mm); 0% re-resection vs. 15.5% in TER	0% local recurrence in TBR group	PNI: 13.3%, LVI: 6.7%	Local recurrence: 0% in TBR, 15.6% in TER	Functional outcomes not detailed	Not reported
Wang et al. (2020) [29]	Taiwan	Cold instruments + IOUS	15	All deep margins >5 mm after IOUS guidance	None	None	No local recurrence	Not reported	Not reported
Kimoto et al. (2017) [22]	Japan	CO_2_ laser	31	28 negative margins, 1 re-resected	None	2 cervical metastases	Local recurrence: 0%	Low VAS pain; resumed soft diet by day 2	1 case (3.2%)
Luukkaa et al. (2002) [24]	Finland	Nd:YAG laser	35	All histologically radical	1 local, 4 regional recurrences	Not reported	Recurrent: 5/35 (local = 1/35, regional = 4/35)	Good tongue function; normal diet resumed	3 cases (8.6%)
Nilsson et al. (2022) [26]	Sweden	Ultrasound-guided resection	110 (34 US-guided, 76 control)	Lower rate of positive deep margins in US group (5.9% vs. 17.1%)	Not reported	Not reported	Not reported	Not reported	Not reported
Metternich et al. (2002) [25]	Germany	Harmonic scalpel	36 (25 tongue SCC)	All margins R0	Not reported	Not reported	No recurrences	Early oral feeding; no infections	Not reported
Gauthier et al. (2010) [20]	Canada	Cold steel (Mohs-like)	12	All final margins negative; 3 required re-resection	None	None	0% recurrence (mean 21 months)	Split graft in 1 case; no major complications	Not reported
Grant et al. (2021) [21]	USA	Transoral laser microsurgery	59	2 positive margins (3%)	Local control 90% at 5 years	Not detailed	4 recurrences; all salvaged	FOSS score unchanged; 8% feeding tube	Not reported
Frame et al. (2004) [19]	USA	CO_2_ laser (post-radiation cases)	21	All negative margins	Local recurrence 29%	Most with prior RT	6 recurrences	Not detailed	1 case (4.8%)
Pons et al. (2012) [9]	France	Harmonic scalpel	17	All R0; avg. margin 16 mm	None reported	Not reported	None	Not reported	Not reported
Rao et al. (2014) [27]	India	Ultrasonic vs. monopolar cautery	60	Wider margins with ultrasonic (deep: 11.3 mm vs. 9.1 mm)	Not reported	Not reported	Not reported	Not reported	Not reported
Tahim & Sadiq (2020) [28]	UK	Electrocautery + scaffold	5	All margins clear	None reported	Not reported	No recurrence at 13 weeks	Good mobility; soft diet by 4 weeks	Not reported
Limongelli et al. (2020) [23]	Italy	Cold steel + bipolar cautery	88	76% negative, 17% close, 7% positive	14.8% local, 17% regional	Margins significantly associated with recurrence	5-year DSS: 81.8%	Not reported	7 cases (7.9%)
Cristall et al. (2020) [18]	Canada	CO_2_ laser	28	17.9% positive deep margins; all mucosal negative	21.4% (all with close/positive deep margins)	Not reported	Recurrence interval: 3-18 months	14% required PEG; no standard scales	Not reported

The harmonic scalpel demonstrated reliable oncologic clearance in studies reporting margin status. In the prospective study by Pons et al., all 17 (100%) patients achieved R0 resection with an average margin of 16 mm. Histologic evaluation confirmed preservation of margin clarity despite a confined thermal injury zone averaging 8 mm [9]. Metternich et al. similarly reported histologically negative margins in all resections using the harmonic scalpel, though cautioned that thermal effects limited microscopic detail within the 0.5-1.0 mm peripheral zone [25].

Ultrasonic dissection was associated with larger histopathological margins compared to monopolar electrocautery. Rao et al. found significantly greater mean margin widths at anterior, lateral, and deep planes in the ultrasonic group, with deep margins averaging 11.3 mm versus 9.1 mm in the monopolar group [27]. No intraoperative frozen section was used. The precision of ultrasonic dissection was echoed in Nilsson et al.’s ultrasound-assisted cohort, where the rate of positive deep margins (≤2.2 mm) was significantly lower than the control group (5.9% vs. 17.1%, p = 0.027) [26].

Intraoperative ultrasound also showed promising results in enhancing margin adequacy. Wang et al. reported a strong correlation (r = 0.87) between intraoperative ultrasound measurements and histological deep margin assessments [29]. Notably, all 15 patients had deep margins >5 mm on final pathology, and in 3 (20%) cases, ultrasound findings prompted intraoperative deepening of the resection.

Cold steel dissection was associated with consistently clear margins in clinical use. Gauthier et al. employed cold instruments in a Mohs-like technique for 12 patients, achieving complete negative frozen margins in all cases, with thicknesses of 1-2 mm in the first half and 5 mm in the latter [20]. Re-resection was performed in three (25%) patients due to initial positivity or concern over inadequate clearance. In a larger retrospective cohort, Limongelli et al. reported that 67 (76.1%) of patients had negative margins following cold steel resection, while 15 (17.0%) were close (<5 mm) and 6 (6.8%) were positive, despite the routine use of intraoperative frozen section [23].

Electrocautery alone was associated with acceptable margin status in selected cases. Tahim & Sadiq reported clear margins in all five (100%) patients undergoing anterior glossectomy, though margin width was not quantified and comparative data were lacking [28]. In contrast, comparative analysis by Rao et al. showed that monopolar cautery produced consistently narrower margins than ultrasonic tools, especially in the deep plane [27].

Thermal Damage and Histological Artefact

Thermal artefact was a prominent concern in studies utilizing heat-generating devices. The harmonic scalpel produced a relatively narrow and predictable thermal injury zone. Pons et al. noted that tissue architecture remained interpretable beyond 1 mm from the resection edge [9]. Similarly, ultrasonic dissection was associated with minimal lateral thermal damage and improved clarity of histological margins compared to monopolar cautery, as qualitatively reported by Rao et al. [27].

Metternich et al. found that histological interpretation was impaired within 0.5-1.0 mm of the resection line when using the harmonic scalpel [25]. While laser resections generally maintained margin interpretability, Cristalli et al. highlighted that positive deep margins were not intraoperatively addressed due to the absence of frozen section analysis in that tissue plane [18].

Cold steel dissection universally avoided thermal artefacts, allowing optimal microscopic interpretation. Gauthier et al. demonstrated that even with narrow margins (1-2 mm), cold scalpel resection combined with frozen section assessment enabled precise intraoperative decision-making [20]. Limongelli et al. employed intraoperative frozen section in all patients, contributing to improved margin control, though the study did not quantify thermal artefact directly [23].

Electrocautery was associated with variable artefact, though this was not systematically quantified in any included clinical study. Thus, while all tools are capable of achieving histologically negative margins, cold instruments and ultrasonic/harmonic devices minimize the risk of diagnostic artefact. CO_2_ laser remains effective but necessitates careful interpretation of deep margins due to its limited tactile depth control.

Recurrence and Local Control

Local recurrence rates differed substantially depending on the clinical context and tool used. Among patients treated primarily with transoral laser microsurgery (TLM), local control was generally high. Grant et al. reported a 5-year local control rate of 90%, with only four (6.8%) recurrences among 59 patients [21]. Importantly, all patients with local recurrence were successfully salvaged. Choi et al., in a prospective comparison of two surgical approaches, observed significantly lower local recurrence in patients undergoing transoral bisected resection (TBR) versus conventional en bloc resection (TER), with rates of 0% (0 patients) and 15.6% (12 patients), respectively (p = 0.037) [17]. The improved control was attributed to deeper margins achieved using a harmonic scalpel and Colorado Bovie tip combination.

Cristalli et al. observed a local recurrence rate of 21.4% (6 patients) following CO_2_ laser resection, with all cases occurring in patients with close or positive deep margins [18]. Similarly, Limongelli et al. reported a 14.8% (13 patients) rate of local recurrence and a 17.0% (15 patients) rate of regional recurrence. Margin status was significantly associated with recurrence in their series, affirming its prognostic value [23].

In patients previously treated with radiotherapy, CO_2_ laser surgery yielded lower control rates. Frame et al. noted a local recurrence rate of 29% (6 patients), despite negative margins, with the majority occurring in previously irradiated fields [19]. Kimoto et al., by contrast, reported no local recurrences among 31 (100%) patients with early-stage cancer, suggesting that laser glossectomy remains oncologically sound in treatment-naïve patients [22].

Ultrasound-assisted resections demonstrated improved margin adequacy, but long-term recurrence data remain limited. Nilsson et al. did not report oncologic outcomes, while Wang et al. noted no local recurrences in their small prospective series, though median follow-up duration was not specified [26,29].

Pons et al. and Rao et al., despite achieving wide margins with ultrasonic and harmonic dissection, respectively, did not provide long-term recurrence outcomes [9,27]. However, the absence of early local failures suggests oncologic feasibility. Gauthier et al. reported no recurrences (0%) in their 12-patient cohort over a mean follow-up of 21 months, although the authors acknowledged that longer-term data are needed [20].

Electrocautery-based resections were associated with short-term disease control in small cohorts. Tahim & Sadiq reported no recurrences within a median follow-up of 13 weeks, though this duration is insufficient to draw conclusions [28].

Some studies also reported regional recurrence. Luukkaa et al. documented four (11.4%) neck recurrences among 35 patients treated with Neodymium:YAG laser, primarily in T1-T2N0 tumors, of whom one died from disease progression [24]. Kimoto et al. observed two cases of delayed cervical metastasis despite negative primary margins, raising concern for occult nodal spread even in early-stage disease [22].

Functional Outcomes

Functional outcomes were most thoroughly evaluated in studies employing TLM. Grant et al. documented preserved swallowing and speech in the majority of patients, with FOSS scores remaining at baseline in long-term survivors [21]. Feeding tube dependence was limited to 8% (5 patients), exclusively among patients receiving adjuvant radiotherapy. Tracheostomy, performed in over one-third of cases (19 patients), was reversed in all but one patient.

Choi et al. did not formally assess functional recovery but noted no significant morbidity associated with the bisected resection approach [17]. In contrast, Kimoto et al. reported early return to oral intake by postoperative day two and minimal pain following CO_2_ laser resection [22]. Similarly, Carruth JA observed rapid resumption of feeding and good functional recovery in 87 (100%) patients undergoing laser glossectomy, with most discharged by day two [16].

Cristalli et al. reported that 4 (14%) patients required PEG tubes postoperatively, though no formal functional scales were applied [18]. The majority resumed normal swallowing and speech. Gauthier et al. described excellent functional outcomes following cold steel resections, with early return to oral feeding and no long-term deficits [20].

Postoperative pain and analgesic requirements were favorably reported in laser-based resections. Carruth JA documented that nearly half of the patients required no analgesia, and few required opioids [16]. Kimoto et al. reported VAS scores between 0 and 2 at two weeks postoperatively, with all patients discontinuing analgesics by one month [22].

Electrocautery and ultrasonic approaches were less frequently evaluated with respect to function. Tahim & Sadiq observed good oral intake and tongue mobility within six weeks postoperatively, though without standardized assessments [28]. Limongelli et al. did not report formal functional outcomes, though the low complication rate suggests acceptable recovery [23]. No studies employing the harmonic scalpel or intraoperative ultrasound included formal evaluation of speech or swallowing outcomes.

Length of hospital stay was infrequently reported, but appeared shortest in laser-treated patients. Carruth JA noted that most patients were discharged by postoperative day two [16]. Kimoto et al. similarly described early discharge and return to oral feeding by day two [22].

Thus, TLM and CO_2_ laser-based resections appear to facilitate favorable functional recovery with minimal pain and short hospital stays. Functional outcomes following newer modalities such as harmonic scalpel, ultrasonic dissection, or intraoperative ultrasound remain underreported and warrant prospective validation.

Hemostatic Performance and Postoperative Complications

Hemostatic efficacy differed according to tool. CO_2_ laser provided effective vessel sealing (up to 1 mm in diameter), resulting in nearly bloodless dissections as noted by Carruth JA [16]. Metternich et al. reported a mean intraoperative blood loss of 30 mL in uncomplicated harmonic scalpel resections and up to 110 mL in cases requiring additional ligation [25]. Luukkaa et al. reported minimal bleeding with Neodymium:YAG laser, although one case required hospitalization for delayed hemorrhage [24].

Cold steel techniques, while effective in terms of margin clarity, were associated with increased bleeding compared to cautery. Gauthier et al. acknowledged greater bleeding with scalpel and scissors, though none required transfusion or led to intraoperative instability [20]. Limongelli et al. reported major complications in 4 (4.5%) patients, including hemorrhage and wound dehiscence, and minor complications in 11.3% [23].

Postoperative complications were uniformly low across studies. Grant et al. reported minor postoperative bleeding in 3 (5% ) in patients, with no infections or aspiration events [21]. Frame et al. and Kimoto et al. reported no wound breakdowns or infections, even in irradiated or anticoagulated patients, respectively [19,22]. Cristalli et al. observed only minor wound-related issues with no major complications [18]. Tahim & Sadiq observed no complications related to electrocautery or scaffold use [28]. Pons et al. and Rao et al. did not provide structured reporting on adverse events [9,27].

In terms of perioperative mortality, CO_2_ laser resection was associated with a rate of 3.2% (1 patient) [22] to 4.8% (1 patient) [19], while Cold steel with bipolar cautery was associated with a rate of 7.9% (7 patients) [23]. Meanwhile, Nd:YAG laser was associated with a death rate of 8.6% (3 patients) [24].

Discussion

The assessment of surgical margins in tongue cancer resection remains a cornerstone of oncologic adequacy and prognostic evaluation. This systematic review synthesizes the evidence on how resection tools-ranging from cold steel scalpel and electrocautery to CO_2_ laser, harmonic scalpel, ultrasonic dissectors, and intraoperative ultrasonography-impact margin status, histological interpretability, recurrence risk, functional outcomes, and perioperative morbidity. The findings reveal substantial heterogeneity in outcomes across instruments, influenced by both the physical characteristics of each tool and the clinical context in which they are employed.

Margin Status and Histological Integrity

Attaining histologically negative margins remains essential for reducing the risk of local recurrence and optimizing disease-specific survival (DSS). However, the definition of an "adequate" margin-particularly in tongue cancer-has long been debated. While a 5 mm clearance has traditionally been advocated, recent evidence suggests a more nuanced interpretation that accounts for tumor biology and anatomical constraints [30]. In this review, both ultrasonic and harmonic tools yielded wide and consistent deep margins, often exceeding 10 mm, with minimal artefactual distortion. Pons et al. and Rao et al. demonstrated that these modalities preserved tissue architecture without compromising hemostasis, suggesting they may offer a favorable balance between oncologic clearance and functional preservation [9,27].

In contrast, CO_2_ laser resections consistently achieved negative margins but introduced challenges in histopathologic interpretation. Multiple studies, including Frame et al. and Kimoto et al., noted pseudodysplastic artefacts and thermal distortion that obscured epithelial and subepithelial transitions [19,22]. These observations align with recent work by Bellini et al., who found that while laser excisions offer precision and favorable healing, they often require pathologists experienced in distinguishing true dysplasia from heat-related changes [31]. This is particularly relevant in narrow-field resections or in cases of carcinoma in situ, where epithelial changes drive surgical decision-making [32].

Cold steel dissection continues to serve as the histological gold standard. In our review, studies using scalpel-based resections [20] reported excellent margin interpretability and consistent negative margins. However, increased intraoperative bleeding, as echoed in the literature [9,19,21], and the lack of inherent coagulative properties limit its use in modern transoral resections, particularly when operative field visualization is critical.

Histopathologic Artefact and Margin Clarity

One of the most compelling findings of this review is the impact of thermal artefact on margin interpretability. Devices such as the CO_2_ laser, although precise, introduce consistent epithelial distortion that can mimic dysplasia or mask residual tumor. The implications of such artefacts are twofold: they may either falsely reassure surgeons or trigger unnecessary adjuvant therapy. Seoane et al., although excluded from the final synthesis due to non-human data, highlighted these artefacts with compelling histologic clarity [10]. Importantly, their observations are echoed by prior research, which argues that even subtle artefactual changes can lead to diagnostic ambiguity [33-36].

Tools like the harmonic scalpel, which operate at lower temperatures (~80°C), appear to mitigate this issue. Pons et al. and Metternich et al. both documented clean margins with preserved tissue planes beyond 1 mm from the cut edge [9,25]. However, even these tools were not free from minor thermal distortion, suggesting that no modality is entirely artefact-free.

Oncologic Outcomes and Recurrence Patterns

The quality and interpretability of surgical margins directly influence oncologic performance. While most studies reported high local control with negative margins, recurrences still occurred, especially in previously irradiated or advanced-stage tumors. This aligns with de Koning et al. [33] and Brennan et al. [36], who emphasized that recurrence is multifactorial, affected by tumor thickness, lymphovascular invasion, perineural invasion, and anatomical site.

Tools achieving wider and more consistent margins (particularly harmonic and ultrasonic devices) may reduce recurrence through improved clearance, although confirmatory data are limited. Grant et al. demonstrated a 90% five-year local control rate with TLM, comparable to meta-analytic benchmarks for early-stage OTSCC [21]. Conversely, Frame et al. and Kimoto et al. [19,22] reported poorer outcomes in previously irradiated fields, underscoring the role of tissue biology in determining success.

Ultrasound-assisted resections further improved deep-margin accuracy. Wang et al. found strong concordance (r = 0.87) between intraoperative ultrasound and histologic depth, allowing real-time resection adjustment [29]. Similarly, Noorlag et al. advocated for image-guided resection to enhance margin adequacy [37]. Nevertheless, the threshold for an “adequate” deep margin remains debated; emerging evidence [38] suggests that required clearance depends on individual tumor behavior rather than a fixed numeric cutoff.

Functional Outcomes and Complication Profile

Functional recovery following tongue resection is increasingly recognized as a determinant of quality of life and long-term survivorship. Our findings reaffirm that TLM is associated with excellent functional outcomes, particularly in early-stage disease. Grant et al. demonstrated that TLM preserves swallowing and speech with minimal need for long-term tracheostomy or enteral support [21]. These outcomes are corroborated by Cheng et al., who observed that smaller, precisely targeted resections correlate with lower FOSS and VAS scores, denoting superior functional preservation [7].

Harmonic scalpel and ultrasonic dissectors, while less extensively studied in this context, appear to yield similarly favorable outcomes, at least in the short term [7,18]. The minimal lateral thermal spread and precise soft-tissue handling of these tools may underlie the preserved neuromuscular integrity observed in the few functional reports available. However, prospective evaluations using validated tools such as the MD Anderson Dysphagia Inventory (MDADI) or Eating Assessment Tool-10 (EAT-10) remain scarce and are strongly recommended in future research [39,40].

Electrocautery, particularly monopolar, appears inferior in both margin quality and histological clarity. Thermal artefact is often pronounced and less predictable, raising concerns about the reliability of final pathology reports. As Carnicelli et al. caution, tools that create variable tissue interfaces may increase the likelihood of both false-positive and false-negative margin assessments, which in turn can influence adjuvant treatment decisions [32].

Intraoperative Margin Guidance and Emerging Modalities

A notable theme emerging from both the review and broader literature is the role of intraoperative margin assessment. Frozen section remains widely used, but its limitations (particularly at the deep margin) are increasingly apparent. Brouwer de Koning et al. [34] reported a 40% discrepancy rate between intraoperative assessment and final pathology at the deep margin, even in high-volume centers.

Intraoperative ultrasonography offers a promising alternative. Both Nilsson et al. and Wang et al. demonstrated that ultrasound could accurately predict histological margin width in real time, prompting additional resections in several cases [26,29]. These findings are echoed by de Koning et al., who found that ultrasound enhanced surgeon confidence and reduced the likelihood of close or positive deep margins without prolonging operative time [35]. While limited by operator experience and equipment availability, intraoral ultrasonography may become an essential adjunct in resection planning, particularly in anatomically constrained sub-sites like the base of the tongue.

These findings must be interpreted in light of the moderate-to-high risk of bias identified across included studies, most of which were single-arm retrospective series. Consequently, findings indicating favorable outcomes for specific tools, such as the harmonic scalpel, should be regarded as hypothesis-generating rather than definitive.

Clinical Implications

From a clinical standpoint, tool selection for tongue cancer resection should be individualized according to tumor depth, anatomic location, prior treatment, and available expertise. Based on current evidence, a pragmatic framework can be proposed: (1) Superficial, early-stage lesions-favor CO_2_ laser or TLM for precision and rapid recovery; (2) Moderately infiltrative or vascular tumors-consider harmonic or ultrasonic devices to balance margin width with minimal artefact; (3) Deep or fibrotic lesions, or previously irradiated fields-cold steel remains optimal for margin interpretability, possibly complemented by electrocautery for hemostasis; and (4) Anatomically complex or high-risk deep margins-integrate intraoperative ultrasonography to guide real-time margin extension. This tiered approach, however, is based on observations from poor-to-good evidence and should be treated as hypothesis-generating, requiring further validation by future research.

Strengths and Limitations

Strengths of this review include comprehensive multi-database searching, strict inclusion criteria, and stratification by resection tool. It is the first synthesis to integrate histopathological, functional, and technological dimensions of tongue cancer surgery. However, this study is not without limitations.

This review is inherently limited by heterogeneity in study design, outcome reporting, and follow-up duration. The absence of randomized comparisons, particularly for newer tools such as the harmonic scalpel and intraoperative ultrasonography, precludes definitive conclusions about oncologic equivalence. Moreover, very few studies applied standardized criteria for functional outcomes, and even fewer included patient-reported quality-of-life metrics. These deficiencies reflect a broader gap in surgical oncology literature, where technical outcomes often eclipse functional and patient-centered endpoints.

The current evidence base is limited by inherent bias in study design. Most available data originate from small retrospective case series or non-comparative cohorts, often performed by single high-volume teams. Selection bias may therefore favor specific instruments, and publication bias could overrepresent favorable outcomes. Furthermore, tools such as the harmonic scalpel, while demonstrating promising histologic and hemostatic profiles, have not yet been evaluated in adequately powered longitudinal cohorts, leaving their long-term oncologic performance uncertain

Additionally, due to the paucity of studies within each tool subgroup for most outcomes, meta-analysis was not feasible. At least three comparable studies per tool-outcome pairing are required for reliable pooled estimates, which were not met in this dataset.

## Conclusions

Our review highlights that resection tool choice significantly influences the histologic quality of surgical margins, with important implications for recurrence risk, functional outcomes, and therapeutic decision-making. While CO_2_ laser and TLM remain standards in early-stage disease, harmonic and ultrasonic tools offer promising oncologic and histological profiles. Emerging adjuncts such as intraoperative ultrasound may further refine resection accuracy. Future research should prioritize prospective, tool-specific comparisons incorporating oncologic, functional, and quality-of-life outcomes, guided by standardized assessment frameworks.

## Appendices

**Table 4:** Detailed search strategy employed in the database search No. number of the search query; #1: search query number 1; #2: search query number 2; #3: search query number 3; #4: search query number 4; #5: search query number 5; #6: search query number 6; #7: search query number 7; #8: search query number 8; tiab: title/abstract filter; AB: abstract; TITLE-ABS-KEY: title, abstract, and keyword

No.	Search query	Results
PubMed		
#1	glossectomy[tiab]	1096
#2	Tongue[tiab]	53615
#3	(cancer[tiab] OR carcinoma[tiab] OR neoplasm[tiab])	2980678
#4	#2 AND #3	12682
#5	#4 OR #1	13297
#6	Margin*[tiab]	272493
#7	scalpel OR laser OR harmonic OR electrocautery OR diathermy OR kinfe OR bovie OR ligasure OR electrosurgery OR cautery OR device OR coagulation OR monopolar	3451033
#8	#5 AND #6 AND #7	131
Scopus		
#1	TITLE-ABS-KEY (glossectomy)	3232
#2	TITLE-ABS-KEY (Tongue)	113454
#3	TITLE-ABS-KEY (cancer) OR TITLE-ABS-KEY (carcinoma) OR TITLE-ABS-KEY (neoplasm)	5612102
#4	#2 AND #3	27781
#5	#4 OR #1	29219
#6	TITLE-ABS-KEY (Margin*)	841898
#7	ALL (scalpel) OR ALL (laser) OR ALL (harmonic) OR ALL (electrocautery) OR ALL (diathermy) OR ALL (kinfe) OR ALL (bovie) OR ALL (ligasure) OR ALL (electrosurgery) OR ALL (cautery) OR ALL (device) OR ALL (coagulation) OR ALL (monopolar)	12258592
#8	#5 AND #6 AND #7	212
Web of Science		
#1	AB=glossectomy	783
#2	AB=Tongue	58050
#3	AB=cancer OR AB=carcinoma OR AB=neoplasm	2606670
#4	#2 AND #3	10428
#5	#4 OR #1	10856
#6	AB=Margin*	594274
#7	ALL=scalpel OR ALL=laser OR ALL=harmonic OR ALL=electrocautery OR ALL=diathermy OR ALL=kinfe OR ALL=bovie OR ALL=ligasure OR ALL=electrosurgery OR ALL=cautery OR ALL=device OR ALL=coagulation OR ALL=monopolar	4116184
#8	#5 AND #6 AND #7	52
